# Can social isolation alleviate symptoms of anxiety and depression disorders?

**DOI:** 10.3389/fpsyt.2025.1561916

**Published:** 2025-04-16

**Authors:** Amanda Peçanha, Bruna Silveira, Thomas E. Krahe, Jesus Landeira Fernandez

**Affiliations:** ^1^ Department of Psychology, Pontifical Catholic University of Rio de Janeiro, Rio de Janeiro, Brazil; ^2^ Departamento de Psicologia, Pontifícia Universidade Catolica do Rio de Janeiro, Rio de Janeiro, Brazil

**Keywords:** social isolation, social behavior, anxiety disorder, depression disorder, emotional dysregulations, avoidance

## Abstract

This study examines the complex interplay between social behavior and mental health, focusing on the neurobiological mechanisms underlying human interactions and their alterations associated with anxiety and depression disorders. These conditions are characterized by heightened threat perception, pervasive worry, physiological responses, emotional dysregulation, and maladaptive behaviors. Through narrative review, this study examines both aspects, addressing social isolation as both a risk factor and an avoidance behavior that may provide temporary relief but ultimately perpetuate the clinical condition in the long term. The findings offer valuable insights for clinical practice, emphasizing interventions that enhance cognitive flexibility to foster stable and supportive interpersonal relationships.

## Introduction

Humans, like other animal species, develop primarily through social interactions across their lifespan (1, 2). Within this framework, the absence of socialization can lead to stress, exerting a profound impact on both physical and mental health (2, 3). Research suggests that the tendency toward isolation can arise from a combination of social, psychological, and neurobiological factors (4–7). While social interactions shape individuals’ self-perceptions and influence their motivation to engage with others, intrinsic characteristics such as personality traits, emotional regulation, and stress responses also play a significant role in determining social withdrawal (8, 9). In this process, the interpretations and perceptions individuals form about themselves through their social interactions directly influence their desire to connect with others, reinforcing patterns of either engagement or avoidance (8).

The dynamics of social interactions are particularly complex for individuals with mental disorders, as these conditions often amplify distortions in reality perception (1, 10). Anxiety disorders, among the most common mental health conditions worldwide, are characterized by excessive fear, worry, and avoidance behaviors triggered by perceived threats (11–14). These disorders often lead to hypervigilance, impaired emotional regulation, catastrophic thinking, physical symptoms such as muscle tension and gastrointestinal disturbances, as well as difficulty concentrating and sleep problems (15–17). Similarly, depressive disorders are marked by a persistent sad, empty, or irritable mood lasting at least two weeks, often accompanied by anhedonia, or the loss of interest and pleasure in previously enjoyable activities (12). Both conditions result in emotional and cognitive impairments that affect daily functioning, with symptoms influenced by genetic predisposition and social factors such as loss, trauma, and chronic stress (11, 18–20). Notably, these disorders frequently co-occur, with approximately 50–60% of individuals diagnosed with an anxiety disorder, particularly generalized anxiety disorder (GAD), also meeting the criteria for depression (18, 21–25). This overlap is attributed to shared genetic vulnerabilities, though anxiety disorders are more influenced by environmental factors, while depression disorders are more closely linked to genetic predisposition (18). Their co-occurrence leads to greater symptom severity, increased functional impairment, and a more chronic and treatment resistant course (17; (16, 26). Anxiety disorders, like depressive disorders, also induce changes in cognitive processing (7). With that said, when these two disorders are comorbid, they can amplify subjective social isolation and the perception of emotional disconnection in relationships, which is one of the dimensions most associated with mental disorders, particularly depression (7, 27). Due to the inherent complexity of mental disorders and social isolation, establishing a causal and sequential relationship between them presents a significant challenge.

Social isolation and mental disorders, such as anxiety and depression disorders, exhibit a complex bidirectional relationship. In some cases, a lack of social interactions may serve as a predisposing factor for the development of these disorders, exacerbating stress responses and impairing emotional regulation. Conversely, individuals with anxiety and depression disorders often struggle to maintain interpersonal relationships, which can lead to a progressive pattern of social withdrawal. This review examines both aspects, addressing social isolation as both a risk factor and an avoidance behavior that may provide temporary relief but ultimately perpetuate the clinical condition in the long term.

## Methods

This narrative review was conducted to integrate findings on the relationship between social isolation, anxiety, and depressive disorders, with a particular emphasis on the underlying neurobiological mechanisms of social interactions and their alterations in affected individuals. The literature selection included empirical studies, systematic reviews, and meta-analyses drawing from widely recognized databases such as PubMed, Scopus, PsycINFO, and Web of Science. References published between 1965 and 2024 were analyzed, using key search terms such as “social isolation,” “social behavior,” “social brain,” “anxiety and depression disorders,” “emotional dysregulation,” and “avoidance”. The selection process focused on studies that provide relevant insights into social behavior and the impact of anxiety and depressive disorders on social interactions. Conversely, studies not directly related to the research theme were excluded. This review was structured into distinct thematic sections to provide a comprehensive and integrative perspective. First, the importance of social interactions is discussed, highlighting their role in emotional well-being and physiological stress regulation. The review then explores the neurobiological foundations of social behavior and social isolation, focusing on brain circuits involved in social processing. A dedicated section further examines the role of key neurotransmitters serotonin, dopamine, and oxytocin in modulating social behavior. Following this, the review outlines the impact of anxiety and depression disorders on the neurobiology of social behavior, emphasizing how these conditions alter emotional processing and social perception. Additionally, it explores the interaction between serotonin, dopamine, and oxytocin within the context of anxiety and depressive disorders. Finally, the review addresses the intersections between emotion, social cognition, and emotional regulation, emphasizing their significance in understanding interpersonal difficulties associated with these disorders.

## The influence of social interactions on anxiety and depression disorders

Humans are inherently social, driven by a fundamental need for belonging that influences their thoughts, emotions, and behaviors (9). While cultural variations exist, humans generally seek stable, lasting relationships over fleeting connections (28). From an evolutionary perspective, forming enduring bonds is vital for survival and reproductive success, contributing to species preservation (29, 30). Adults within groups have a higher likelihood of survival due to access to support in risky situations and care during illness (28, 29).

Beyond survival, social connections foster health and resilience within communities (28). Individuals who build relationships often experience positive emotions such as happiness, empathy, and a sense of belonging which enhance their quality of life and support long-term emotional well-being (10, 31).

Emotional experiences, being deeply personal, play a fundamental role in socialization by influencing group dynamics and signaling risks, belonging, or exclusion among peers (32). Although emotions are unique to each individual and may sometimes be suppressed, they remain a crucial motivating factor that is reflected in observable behaviors and is subject to the influence of interpersonal interactions (32, 33). Social interactions, in turn, amplify these emotions, uniting individuals in shared experiences—whether joyful or challenging, such as fear (32, 34). Innate skills, such as empathy and emotional contagion are essential for promoting a sense of belonging within groups, as they facilitate emotional connections between individuals (32, 35).

At a cognitive level, adaptive thoughts are those linked to a sense of belonging and the pursuit of secure bonds within a group (28). Human cognition, characterized by its sophistication and unique capacity for socially contextualized learning and emotional sharing, underpins our sociability (36, p. 14-35). This intricate network of cognitive interactions, refined through evolution, enables self-evaluation and peer assessment while also exposing us to both cognitive rigidity and flexibility (36, p. 14-35; 32).

The ability to form interpersonal connections is essential for securing social support (37). However, individuals lacking secure relationships are more vulnerable to developing psychological disorders, such as anxiety and depression disorders, which can severely affect mental and physical health (7, 37, 38). Perceived loneliness, rooted in the psychological sensation of not belonging, significantly activates the alert system, triggering stress responses that can lead to physiological, cognitive, and immunological changes, thereby increasing risks to both physical and mental health (7, 27, 38, 39). Furthermore, difficulties in interpreting, expressing, and experiencing emotions can hinder social interactions, resulting in reduced support and increased misunderstandings (40).

As several studies indicate, a lack of socialization is strongly associated with an increased risk of developing anxiety and depression disorders (7, 27, 39). However, preexisting psychological factors may contribute to social isolation, while inadequate social support can further compromise mental health, creating a bidirectional cycle (41, 42). Moreover, the development of psychological disorders is influenced by a complex interplay of factors, including genetic predisposition, traumatic experiences, chronic stress, neurobiological dysregulation, and environmental influences (43, 44).

Emotional and cognitive adjustment difficulties commonly observed in anxiety and depression disorders impair individuals’ adaptive capacity, significantly affecting their interpersonal relationships (15, 16, 45). The subjective perception of being understood, respected, and emotionally supported plays a crucial role in determining the extent to which individuals believe their social support network can alleviate depressive symptoms (46). The literature highlights resilience as a key coping mechanism that integrates intrinsic and interpersonal factors, promoting adaptive capacity, fostering more positive responses to adversity, and reducing symptoms of anxiety and depression (47, 48). Relationships characterized by open communication, where individuals feel safe expressing difficulties and receiving support, are considered beneficial as they enhance adaptability to life challenges (47, 48). Furthermore, relationships that encourage acceptance, flexibility, and self-care practices contribute to stress reduction, which is crucial for managing these disorders (47). While social relationships can serve as a protective factor against anxiety and depression symptoms, negative social interactions have the potential to exacerbate psychological distress (47, 49). Higher levels of negative social interactions are significantly associated with increased symptoms of depression and anxiety (49). Interactions with friends and family that convey unavailability, excessive demands, harsh criticism, judgment, and persistent relational conflicts leading to distress and stress have a particularly detrimental impact on mental health (49).

## Neurobiological bases of social behavior and social isolation

Social behavior is regulated by various brain regions and neural circuits, each contributing to different aspects of social and cognitive functioning (50). Research highlights the connectivity between structures involved in social cognition, such as the Default Mode Network (DMN), which supports psychological processes essential for attributing meaning to the mental states of oneself and others- critical during social interactions (51, 52).

The regions comprising the Default Mode Network (DMN) operate in an integrated manner, continuously associating individual and environmental factors to construct shared meanings, thereby facilitating social communication (53, 54). Several of these regions perform self-referential functions, including the medial prefrontal cortex (mPFC), the posterior cingulate cortex (PCC), and the temporoparietal junction (TPJ), which are involved in differentiating the self from others (51, 55). The precuneus is associated with the processing of personal information, while the angular gyrus plays a role in retrieving relevant memories (55). Moreover, the DMN plays a central role in social cognition, anticipating future actions based on past experiences and exhibiting heightened responses to interactions with people compared to objects (51, 55). Evidence suggests that functional connectivity between the superior frontal gyrus and the superior parietal lobule is more pronounced during interactions with close individuals than with strangers (55). Complementarily, studies indicate that the interaction between the DMN and the ventral striatum, mediated by the executive control network (ECN), enhances the value of social rewards derived from close interpersonal relationships, highlighting the role of these connections in modulating social behavior (54, 55). According to the literature, the activation of the (DMN) plays a crucial role in modulating prosocial responses, both behaviorally and emotionally. This process occurs as the DMN integrates internal cognitive states with external environmental cues, enabling the production of socially appropriate reactions (56).

Studies in social neuroscience highlight the role of cortical regions in analyzing complex social behaviors (50). For instance, the anterior insular cortex (AI) interacts with different brain regions depending on the social context, playing a significant role in affective states that range from positive feelings, such as compassion, admiration, and a sense of justice, to negative experiences like pain and disgust in social interactions (57). Due to the adaptive value of social connection, the social pain triggered by perceived rejection or lack of belonging is often likened to physical pain in its ability to signal potential risks (39). In line with this hypothesis, neuroimaging studies reveal the activation of similar brain structures in both physical and emotional pain, including the insula (IA), anterior cingulate cortex (ACC), and dorsal anterior cingulate cortex (dACC) (39, 58, 59).

Humans and other mammals exhibit strong social motivation, driven in part by their capacity for empathetic communication, which is widely considered genetically determined (60). The pro-social tendency to share emotional states and seek support during stress reduces activity in the anterior cingulate cortex (ACC), a region essential for emotional regulation. This process also downregulates activity in the amygdala and the hypothalamicpituitary-adrenal (HPA) axis, aiding stress management (61).

Research suggests that the same neural networks are involved in both the identification and expression of emotions (60). Among these, the amygdala plays a central role in recognizing facial expressions, particularly those associated with fear. It integrates sensory information from environmental stimuli with behavioral, autonomic, and endocrine responses critical for emotional recognition (60–63).

Neural systems that synchronize across individuals have been identified at the cortical level in processes such as understanding actions, processing pain, and recognizing emotions (33, 60). This phenomenon forms the neurophysiological foundation of social cognition through the automatic activation of motor or emotional representations (60). Moreover, patterns of activation and deactivation vary depending on factors such as context and the specific emotions involved (60).

A key component of socialization is the ability to perform executive functions, particularly understanding the mental states of oneself and others (31, 60). The prefrontal cortex (PFC) plays a vital role in regulating social behavior, including inhibitory control, self-regulation, and cognitive flexibility (31, 60, 64). Additionally, the right inferior parietal cortex contributes to the ability to assertively adopt another’s perspective, allowing for the understanding of their experiences without one’s own perceptions interfering in this process (60). These capabilities are essential for developing empathy and social wellbeing, which are deeply connected to the individual’s interactions and environment (60). Interactions among the medial prefrontal cortex, amygdala, and orbitofrontal cortex facilitate the processing of information critical for socialization (63). Similarly, the hippocampus (HPC) plays a key role in social memory, aiding in the discrimination between familiar and unfamiliar individuals, as well as in less complex social behaviors, such as sexual behavior (50, 65).

Oxytocin, a neuropeptide produced in the hypothalamus, is released into both the bloodstream and brain in response to reproduction-related stimuli, such as childbirth, breastfeeding, and sexual activity, as well as during social interactions and stressful situations (66). Its release highlights humans’ ultrasocial nature, demonstrating an intrinsic motivation to connect with others from an early age (67). Moreover, it is well accepted that oxytocin plays a critical role in regulating amygdala activity by reducing hypothalamus and hypothalamic-pituitary-adrenal (HPA) axis activation in stressful situations (68). This regulation promotes more adaptive social behaviors (66, 68). Therefore, oxytocin integrates cognitive processes that favor social interactions in healthy individuals (66). However, its dysregulation has been linked to mental disorders associated with difficulties in forming and maintaining social relationships (19).

In contrast to the neural activations associated with pro-social behaviors, social isolation is linked to the dysregulation of key neural circuits involved in emotional regulation, stress response, and social cognition. Studies indicate that social isolation leads to reduced connectivity between the medial prefrontal cortex (mPFC) and limbic structures, impairing emotional regulation and increasing vulnerability to anxiety and depression disorders (69, 70). This weakened mPFC-limbic connectivity disrupts topdown inhibitory control over amygdala activity, leading to heightened threat perception, increased fear responses, and exaggerated stress reactivity (42, 71). Concurrently, hippocampal dysfunction impairs stress contextualization and cognitive flexibility, which are essential for adaptive coping mechanisms (71). Moreover, hyperactivation of the hypothalamic-pituitary-adrenal (HPA) axis further exacerbates neuroinflammatory responses and cortisol dysregulation, contributing to long-term impairments (70, 71).

## Serotonin, dopamine and oxytocin modulation of social behavior

The serotonergic system (5-HT) plays a relevant role in fundamental processes vital for survival and species preservation, including mood regulation, reproduction, feeding, sleep, pain perception, temperature control, learning, memory, and social behaviors (72). Social interactions are strongly influenced by serotonergic activity, which is highly responsive to environmental stimuli and context-dependent factors (72, 73). Emotionally secure and socially enriched environments tend to promote more adaptive, sociable, and resilient behaviors in the face of challenges (72, 73). Conversely, stressful environments can disrupt the serotonergic system, reducing mood and impairing social behaviors (72, 73). The role of serotonin is essential in the maintenance of emotional well-being, promoting more reflective and less impulsive behaviors that are conducive to social contexts (74).

Dopamine, another neurotransmitter of significant importance, not only facilitates but is essential for motivation and gratification in positive social interactions (61, 64). This neurotransmitter plays a critical role in the modulation of social behaviors by systematically processing information and valences that enhance social learning (75). In this manner, social experiences can alter dopaminergic activity, which may subsequently lead to either social cohesion or social withdrawal (75). Furthermore, the functional interaction between serotonin and dopamine also encompasses cognitive functions, and alterations in their levels are associated with mental disorders that impact social relationships (74, 75).

Oxytocin, a neuropeptide produced in the hypothalamus, is released into both the bloodstream and brain in response to reproduction-related stimuli, such as childbirth, breastfeeding, and sexual activity, as well as during social interactions and stressful situations (66). Its release highlights humans’ ultrasocial nature, demonstrating an intrinsic motivation to connect with others from an early age (67). Moreover, it is well accepted that oxytocin plays a critical role in regulating amygdala activity by reducing hypothalamus and hypothalamic-pituitary-adrenal (HPA) axis activation in stressful situations (68). This regulation promotes more adaptive social behaviors (66, 68). Therefore, oxytocin integrates cognitive processes that favor social interactions in healthy individuals (66). However, its dysregulation has been linked to mental disorders associated with difficulties in forming and maintaining social relationships (19).

## Neurobiology of anxiety and depression disorders

Integrating environmental stimuli with stored memories is a dynamic and essential cognitive process that enables individuals to interpret and respond to external inputs. This process is shaped not only by beliefs and emotions but also by the unique ways individuals perceive and process stimuli, resulting in highly personalized patterns of interpretation and reaction (53). These patterns are further influenced by personal experiences, contextual factors, and prior knowledge, which collectively modulate cognitive and emotional responses to the environment (53).

At the neural level, the Default Mode Network (DMN) serves as a central system for orchestrating this integration. The DMN enables seamless transitions between introspection and environmental engagement, supporting key cognitive processes such as self-referential thinking, episodic memory retrieval, and social cognition (51, 76). It helps individuals construct coherent internal narratives by integrating memory, language, and semantic representations, essential for imagining future scenarios and reflecting on past experiences (51, 77). Furthermore, the DMN interacts dynamically with other brain networks, such as the salience and executive control networks, to balance attention between internal and external demands (51, 76). By harmonizing internal and external information, the DMN plays a pivotal role in emotional regulation, adaptive decision-making, and resilience, highlighting its importance in mental health maintenance (51). In depressive and anxiety disorders, clinical complexity often stems from the interplay of genetic predispositions and environmental stressors, which contribute to their high comorbidity rates (18, 78). The overlapping biological, psychological, and social factors associated with these disorders pose significant challenges for therapeutic interventions, requiring approaches that address both shared and disorder-specific features (78).

Research highlights the significant impact of depressive and anxiety disorders on the DMN, with alterations affecting not only psychiatric symptoms but also social processes, such as the perception and interpretation of emotional expressions (53, 79). These DMN modifications are not exclusive to depression and anxiety disorders, they are also observed in other conditions characterized by social dysfunctions (53, 78, 79).

Regions of the prefrontal cortex (PFC), as key components of the DMN, play a vital role in functions such as self-referential thinking, belief evolution, and emotional identification (79). In individuals with depression disorder, functional deficits in these areas are closely linked to impaired emotional regulation and difficulties in establishing healthy social interactions. Numerous studies have highlighted that one of the key characteristics of depressive disorder is a decrease in motivation, which can lead to social isolation, often considered the primary cause of depression (72, 75). This alteration can be explained by anhedonia, a symptom commonly associated with depression, characterized by the loss of pleasure in activities previously perceived as enjoyable (61, 80). Neuroimaging studies have also revealed that reduced functional connectivity in brain structures involved in reward processing and social cognition, such as the default mode network (DMN), is associated with difficulties in forming social bonds and experiencing pleasure (79). These findings support the hypothesis that social isolation results from neural mechanism alterations related to motivation and reward rather than solely external factors (79). These difficulties can exacerbate social isolation, perpetuating depressive states and reinforcing the cycle of emotional distress and diminished social engagement (53, 79). A decline in social interactions is a key feature of depression, reflected in reduced engagement with social rewards, heightened emotional suffering, and impaired interpretation of social cues (45, 78). These challenges are often associated with reduced intrinsic motivation, increased vulnerability to rejection, diminished collaboration, and avoidance of competitive or demanding social scenarios. This impaired ability to navigate social contexts leads to maladaptive social decisions, perpetuating isolation and worsening depressive symptoms (45). Additionally, a strong vulnerability to social rejection is frequently tied to cognitive distortions in self-evaluation and negative interpretations of social interactions, which reinforce maladaptive emotional responses and sustain the depressive state (16, 45).

A central mechanism contributing to reduced socialization and heightened emotional suffering involves the insula, a brain region critical for integrating internal bodily states with external social and environmental information (45). The insula’s role in creating a holistic emotional perception links it to the experience of emotional suffering in social contexts. Alongside the insula, the amygdala is hyperactivated in individuals with depression and anxiety, driving the heightened processing of negative emotions and reinforcing threat-related responses (15, 45). This hyperactivity also extends to the nucleus accumbens (NAcc), a structure involved in reward processing, further disrupting the balance between emotional regulation and behavioral responses. Dysfunctional communication between subcortical and cortical regions, such as the orbitofrontal cortex (OFC) and medial prefrontal cortex (mPFC), impairs excitatory control, exacerbating emotional dysregulation, and contributing to feelings of disconnection and social withdrawal (15, 45, 81).

Chronic stress, often fueled by negative social interpretations, induces significant physiological and cognitive changes, including mood alterations and impaired information processing (82, 83). The hippocampus, which is closely connected to subcortical regions, plays a critical role in memory encoding and contextualizing emotional experiences (15). In individuals with depression and anxiety disorders, disruptions in hippocampal function are associated with distorted information processing, favoring negative biases and ruminative thinking (15, 16). These dysfunctions are further exacerbated by chronic activation of the hypothalamic-pituitary-adrenal (HPA) axis, leading to prolonged norepinephrine and cortisol release, which undermines cognitive and emotional stability (16, 82, 84). Dysregulation of the HPA axis perpetuates a cycle of stress, emotional dysregulation, and cognitive impairments, commonly observed in anxiety and depression disorders (82, 85).

An increased focus on processing negative emotions may stem from excessive rumination and impaired social cognition (8, 86). Social cognition involves perceiving, interpreting, and responding to social information, and disruption of such cognitive ability often leads to withdrawal from social interactions (8). Although disruptions in brain activities related to negative emotions are particularly prominent in individuals with depression, those with anxiety disorders also show significant impairments in recognizing emotional stimuli, especially a heightened identification with expressions of fear (78).

Emotional tension, a hallmark feature of anxiety, is frequently linked to avoidant and inhibitory behaviors, as well as an increased tendency to perceive obstacles in social and environmental contexts (78). In depression, reduced social motivation primarily results in challenges with forming and maintaining intimate relationships (16, 87). Heightened sensitivity to negative evaluations— of both self and others - further impairs self-worth and decreases the willingness to participate in social interactions (88). However, despite a tendency toward social isolation, engaging in social activities often leads to symptom improvement in these individuals (78).

## Dopamine, serotonin and oxytocin interactions on anxiety and depression disorders

Neurotransmitter-based studies have demonstrated that serotonin deficiency compromises dopamine release, suggesting an impairment in reward processing and anhedonia, both of which are core symptoms of depressive disorders (72, 75, 80). Dysfunction in this interaction may impair the salience of social stimuli, contributing to reduced motivation for social engagement (61, 80).

In anxiety disorders, serotonergic hyperactivity disrupts dopaminergic mechanisms, reinforcing avoidant behaviors (74, 80). Key brain regions implicated in these dysregulations include the prefrontal cortex, nucleus accumbens (NAcc), and amygdala, all of which play a central role in emotional and cognitive regulation (15).

Alterations in oxytocin levels in individuals with anxiety and depressive disorders driven by impairments in brain regions involved in threat perception and emotional regulation, have been linked to increased social withdrawal, social dysfunction and heightened sensitivity to social rejection (68, 89, 90).

## Emotion and social cognition

Individuals respond emotionally based on their experiences and interpretations (19, 30). However, discrepancies frequently emerge between emotional states and their outward expressions, such as speech, behaviors, or facial cues. These inconsistencies stem from the influence of social and clinical factors on the neural and psychological mechanisms that govern emotional processing (68, 91).

Non-verbal emotional expressions, such as facial and bodily cues, are essential for universal communication and group cohesion, enabling adaptation to dynamic environments (19, 30). Verbal communication, meanwhile, plays a crucial role in articulating emotional experiences, minimizing misunderstandings, and fostering closeness between individuals (19, 30). This is particularly important given that subjective interpretations often lead to miscommunication (19, 30).

Expressing emotions, such as sadness, can elicit empathetic responses from others and reinforce a sense of belonging, even without physical proximity, particularly when driven by affection and care (19). The ability to assertively express emotions— whether positive or negative—generally strengthens relationships, in contrast to suppression or indifference, which can weaken connections (19, 92). However, when emotional expressions are misaligned with the social context, they may lead to relational challenges. Therefore, regulating the intensity, duration, and timing of emotional expressions is essential (91, 92). Conversely, some individuals heavily rely on others’ emotional expressions to evaluate their own value or abilities (19). During social interactions, people form self-judgments influenced by the emotions they experience, which in turn shape their social behaviors—whether authentically expressed or modified to seek social acceptance (93).

The ability to manage emotions and behaviors in response to social demands is associated with frustration tolerance, the capacity to cope with fear and anxiety, acceptance of solitude, and enjoyment of social interactions (94). Recent studies suggest that emotional intelligence and social problem-solving skills are governed by a network of shared brain regions (95). These studies also explore the hypothesis that individual differences influence social cognition (95). Dysregulation in these areas is often linked to cognitive dysfunctions, including rigid belief systems and subjective perceptions, which are commonly observed in psychiatric disorders such as anxiety and depression (94–96). Promoting cognitive flexibility can help alleviate negative emotional experiences, such as anger, sadness, anxiety, and hopelessness (94, 96).

Emotions significantly influence cognitive processes, with individual approaches to environmental interaction and information interpretation directly shaping social interactions and group dynamics (97, 98). For effective group cohesion, flexibility in action selection is crucial for balancing individual needs with those of the group, facilitating harmonious relationships (99). As social interactions become increasingly complex, this flexibility requires the development of cognitive skills that enhance information processing, memory retention, and the recognition of signals that improve one’s reputation within a group. These skills are also essential for avoiding harmful interactions, further strengthening social cohesion (100).

The interaction between cognition and emotional responses involves dynamic processes in which contextual, emotional, and individual characteristics modulate emotion regulation (101). Evidence suggests that essential skills for well-being, such as empathy, can influence this interaction in distinct ways, depending on their nature (102). In the study conducted by Thompson et al. (102), cognitive empathy, characterized by the ability to understand others’ emotions without sharing their emotional state, is associated with greater emotional self-regulation. In contrast, affective empathy, which involves sharing another person’s emotional state, tends to elicit more automatic emotional responses, making emotion regulation more challenging. This interaction does not occur in a static manner, demonstrating that in contexts of psychosocial stress and in real-time situations, individuals exhibit cognitive or automatic flexibility when regulating their emotions (103). To achieve this, different strategies, such as cognitive reappraisal, interoceptive awareness, and mindfulness, are employed to adapt to challenging social situations (101). Cognitive reappraisal, in particular, has been consistently shown to engage prefrontal control regions and modulate amygdala activity, as demonstrated by neuroimaging meta-analyses (104). Therefore, this dynamic regulation plays a crucial role in social cognitive processing, particularly in emotion recognition and expression. In this context, cognitive reappraisal facilitates a more accurate interpretation of emotional cues, while interoceptive awareness and mindfulness modulate emotional responses, promoting more adaptive social interactions (101–103).

## Emotional and cognitive regulation

Anxiety and depressive disorders are often marked by changes in emotional and cognitive processes, including heightened emotional suffering, self-criticism, and negative social interpretations (45, 78, 105). These changes are evidenced by avoidant behaviors, as individuals with high emotional sensitivity often evade discomfort, such as anxiety (106). Thus, the way an individual experiences and expresses emotions can profoundly affect their mental health and ability to meet social demands (79, 107, 108).

Emotional dysregulations commonly observed in anxiety and depression disorders may be linked to alterations in neural networks, as outlined in a comprehensive review of the literature (15, 45, 53, 79). These dysregulations manifest as self-referential criticisms, increased vulnerability to rejection, and avoidance of social demands, such as the motivation to cooperate or compete (16, 45). Psychological suffering, exacerbated by diminished cognitive self-control, ineffective coping strategies, and difficulties in cognitive reappraisal, especially in social situations, often leads to hypervigilance and a sense of alienation in individuals with anxiety and depression disorders (39, 107). Hypoactivation of brain regions essential for cognitive reappraisal may explain why these individuals are more likely to resort to suppression, rumination, and avoidance as strategies to reduce the intensity and expression of negative emotions (45, 79, 109–111). Rumination can lead individuals to engage in avoidance behaviors concerning their disturbed mental states. Similarly, suppression does not alter the subjective experience of emotions but rather inhibits their outward expression (109–111). Such coping strategies for managing emotional vulnerabilities are frequently linked to isolation and temporary relief from the perceived demands of social interactions experienced by individuals with anxiety and depression disorders (53, 79). In this regard, the studies conducted by Cacioppo and Hawkley (39) highlight the interplay between the sensation of disconnection associated with the quality of social interactions and cognitive alterations. This suggests that a temporary relief may occur when individuals, as a defensive mechanism, avoid what they perceive as social demands (9, 45, 79).

## Discussion

Empirical evidence suggests that social isolation may heighten vulnerability to anxiety and depressive disorders, as the absence of social interactions is associated with increased stress reactivity and impaired emotional regulation (39). Prolonged isolation can induce dysregulation of the hypothalamic-pituitary-adrenal (HPA) axis, leading to elevated cortisol levels and an exaggerated response to aversive stimuli (84). Furthermore, the lack of social reinforcement may attenuate dopaminergic system activity, thereby compromising motivation and diminishing the hedonic value of social interactions (15, 38).

Another important aspect is that the predominance of negative emotions in anxiety and depressive disorders is associated with increased threat perception, emotional dysregulation, and social withdrawal (7, 45, 105). Preclinical studies using high-anxiety rat models have also demonstrated reduced social interest and increased freezing behavior, further reinforcing the link between anxiety-related traits and social avoidance ( (112). Notably, when these animals are subjected to social isolation, depressive-like behaviors tend to diminish, suggesting a potential protective effect of isolation in certain contexts (113). Moreover, neuroimaging studies in humans highlight structural and functional alterations in the medial prefrontal cortex (mPFC), amygdala, and orbitofrontal cortex (OFC), supporting the notion that impairments in social signal processing may contribute to social withdrawal (15, 45, 53, 79). From a cognitive perspective, increased rumination and heightened attentional bias toward negative stimuli further reinforce subjective social isolation exacerbating avoidant behaviors (7, 27, 38, 45, 78, 86). This paradox may be partially explained by the role of chronic stress in modulating neural circuits involved in social motivation. Persistent hyperactivation of the hypothalamic-pituitary-adrenal (HPA) axis, often observed in individuals with anxiety and depression, can contribute to heightened vigilance and increased avoidance behaviors (82, 84).

Social isolation, when adopted as an avoidance strategy by individuals with anxiety and depressive disorders, may serve as a coping mechanism to mitigate the emotional burden associated with social interactions (45). An alternative hypothesis suggests that dysfunction within the dopaminergic reward system may contribute to reduced motivation for social engagement (79).

Although withdrawal from social interactions may temporarily alleviate the emotional burden in these individuals, clinical interventions should prioritize the promotion of high quality social engagement, addressing both vulnerability to isolation and the challenges individuals face in social contexts. Cognitive Behavioral Therapy (CBT) has long demonstrated efficacy in cognitive restructuring and the modulation of adaptive responses for various mental disorders (114). With advancements in research, studies such as those by McRae and Gross (107) emphasize the integration of cognitive reappraisal and emotional regulation strategies, as utilized in CBT, to enhance social resilience in individuals with anxiety and depressive disorders. Neuroimaging studies highlight the ability of cognitive reappraisal to activate the prefrontal cortex, thereby reducing amygdala hyperactivity and adjusting emotional responses. In clinical practice, graduated exposure, systematic desensitization, and the modification of dysfunctional beliefs are widely implemented to reduce emotional avoidance and enhance coping strategies in anxiety-provoking situations (104, 115–117). Furthermore, integrated with CBT, Mindfulness-Based Stress Reduction (MBSR) and Mindfulness-Based Cognitive Therapy (MBCT) have been increasingly incorporated into the treatment of anxiety and depressive disorders, demonstrating significant structural and functional benefits (118). These interventions contribute to emotional regulation, attentional control, and self-referential processing, facilitating prosocial behaviors (118). Mindfulness practices can be applied individually or in group settings, effectively reducing rumination and enhancing treatment outcomes across different therapeutic modalities.

Emotion-Focused Therapy (EFT), which centers on transforming distressing emotions, facilitates experiential processing and the restructuring of maladaptive emotional schemas (119, 120). This approach emphasizes empathy and experiential interventions, such as emotional evocation techniques, to foster emotional regulation and interpersonal skill development (119, 120). As indicated by several studies, Emotion-Focused Therapy (EFT) has proven to be effective in addressing symptoms commonly observed in anxiety and depression disorders, such as self-criticism and feelings of disconnection, thereby promoting emotional regulation (121, 122) Given that managing worry and emotions represents a primary challenge for individuals with anxiety disorders, Dialectical Behavior Therapy (DBT)—which incorporates emotional regulation techniques, distress tolerance, mindfulness, and interpersonal effectiveness training—has emerged as an effective intervention for enhancing cognitive flexibility and emotional regulation (123). Originally developed for borderline personality disorder, DBT has demonstrated significant benefits in improving cognitive flexibility and emotional control in individuals with anxiety disorders, particularly generalized anxiety disorder (GAD) (123).

Considering the structural and functional alterations common to anxiety and depressive disorders, as well as the challenges associated with their treatment, integrating emotional regulation techniques, cognitive flexibility training, mindfulness, and interpersonal skills development becomes essential for optimizing treatment efficacy. The literature suggests that in cases of comorbidity between anxiety and depressive disorders, greater symptom severity may necessitate a combination of psychological and pharmacological interventions to achieve optimal therapeutic outcomes (18).

## Limitations

Like any narrative review, this study has some limitations that should be considered. While it provides a comprehensive synthesis of the literature on the interplay between social isolation, anxiety, and depressive disorders, it lacks the systematic rigor of meta-analyses, making it susceptible to selection and publication biases. The heterogeneity of study designs, including differences in sample sizes, methodologies, and assessment tools, may limit the generalizability of findings. Additionally, most studies reviewed are cross-sectional, restricting conclusions about causality and the long-term impact of social isolation on mental health. Although this review focuses primarily on neurobiological mechanisms, it is important to acknowledge the role of sociocultural and environmental influences, which require further exploration. Moreover, the potential influence of confounders, including personality traits and pre-existing psychiatric conditions, was not systematically addressed, which may impact the interpretation of results. Nonetheless, these limitations do not diminish the value of this work, highlighting opportunities for future research to incorporate longitudinal studies and a more integrative approach to better understand the complex relationship between social isolation, anxiety, and depression.

## Conclusion

Social relationships are essential for physical and mental health, playing a key role in fostering a sense of belonging - an intrinsic human predisposition. Within this framework, social isolation is widely recognized as a major risk factor for numerous illnesses, such depression and anxiety disorders. While patients with these mental disorders may find temporary relief from social stressors through isolation, their fundamental need for social connection remains critical. An understanding of the neurobiological mechanisms that drive social interactions, as well as the integration of social and personal information is essential for promoting healthy interpersonal relationships in clinical settings. In this regard, assessing the impact of relational difficulties is critical, especially since these challenges frequently emerge from anxiety and depressive disorders. Identifying the neurobiological foundations linking the social and cognitive aspects of these disorders is imperative. Such insights may pave the way to understand the interplay between these factors and support the development of more effective therapeutic interventions. This understanding is particularly important in strengthening the therapeutic alliance between patient and therapist, thereby enhancing treatment outcomes.
